# Clinical and echocardiographic findings in patients with COVID-19 across different severity levels

**DOI:** 10.25122/jml-2023-0206

**Published:** 2023-11

**Authors:** Ihor Hryzhak, Oleksandra Pryshliak, Taras Kobryn, Sergiy Fedorov, Oleksandr Boichuk, Oleksandra Marynchak, Viktoriia Kvasniuk, Andrii Protsyk, Ruslan Miziuk, Andrii Kucher, Marianna Simchych, Lilia Hryzhak, Mariia Kuravkin

**Affiliations:** 1 Infectious Diseases and Epidemiology Department, Ivano-Frankivsk National Medical University, Ivano-Frankivsk, Ukraine; 2 Department of Therapy, Family and Emergency Medicines of Postgraduate Education, Ivano-Frankivsk National Medical University, Ivano-Frankivsk, Ukraine; 3 Department of Airborne Infections of Communal Non-Commercial Enterprise, Ivano-Frankivsk Phthisiatry-Pulmonology Center, Ivano-Frankivsk, Ukraine

**Keywords:** viral infection, heart failure, pulmonary hypertension, risk factor, disease outcome, AV – aortic valve, BMI – body mass index, CHF – chronic heart failure, FC – functional class of chronic heart failure, HF – heart failure, LV – left ventricle, MV – mitral valve, RV – right ventricle, RF – respiratory failure, PA – pulmonary artery, PH – pulmonary hypertension, TV – tricuspidal valve

## Abstract

Cardiovascular pathology can complicate the course of COVID-19. The study aimed to identify echocardiographic abnormalities and key prognostic factors influencing severe and fatal COVID-19 outcomes. This retrospective cohort study included clinical and echocardiogram data from 194 medical records of hospitalized patients with COVID-19: 100 moderate cases, 34 severe cases with favorable outcomes, and 60 severe cases with fatal outcomes. Severe patients with favorable outcomes had greater reductions in left ventricular systolic fraction of left ventricle compared to moderate cases (23.5% *vs*. 7.0%, respectively, p=0.008) and ejection fraction of left ventricle (14.7% *vs*. 3.0%, respectively, p=0.013), grade I diastolic dysfunction of the left ventricle (20.6% *vs*. 8.0%, respectively, p=0.044), and pulmonary hypertension (29.41% *vs*. 10.0%, respectively, p=0.006). Patients with fatal outcomes had a mean age of 67.1±1.51 years, chronic heart failure functional class II (58.3%), hypertension (50.0%), type 2 diabetes (43.3%), and obesity (33.3%). Compared to severe cases but with favorable outcomes, fatal cases had a greater decrease in left ventricular ejection fraction (36.7% *vs*. 14.7%, respectively, p=0.024), various types of myocardial dysfunction (51.7% *vs*. 29.4%, respectively, p=0.037) and a trend towards increased pulmonary hypertension (48.3% *vs*. 29.4%, respectively, p=0.074). Consequently, chronic heart failure class II, reduced left ventricular ejection fraction, various myocardial dysfunctions, and pulmonary hypertension emerged as key cardiac risk factors for severe disease progression and mortality in patients with COVID-19.

## INTRODUCTION

To date, many diseases and pathological conditions have been identified as predictors of severe COVID-19, such as hypertension, coronary heart disease, diabetes, obesity, and age after 60 years [1, 2]. In-hospital mortality rates are higher when COVID-19 is associated with acute kidney injury, chronic heart failure (CHF), and cardiogenic shock [3]. In patients with COVID-19 with severe acute respiratory syndrome, signs of myocardial damage are often observed (63.2%), which are manifested by changes in the ST segment and an increase in the level of troponin. In addition, transthoracic echocardiography reveals a wide range of cardiac abnormalities: global left ventricular (LV) dysfunction, LV diastolic dysfunction II and III grades, local LV wall dyskinesia, right ventricular (RV) dysfunction, and pericardial exudation. Patients with COVID-19 may have the highest mortality risk (31.7%) in the presence of echocardiographic abnormalities and myocardial involvement [4]. A history of heart failure (HF) in patients with COVID-19 is associated with a higher mortality rate than in patients hospitalized for acute HF without COVID-19 [5, 6]. Therefore, this syndrome can be an independent predictor of nosocomial mortality from COVID-19 [7].

The severe form of COVID-19 is closely related to RV dysfunction. Meta-regression studies have shown that RV dysfunction is observed in 20% of patients with COVID-19. It is also a risk factor for all-cause death [8, 9]. Echocardiographic studies revealed RV dilation with or without systolic dysfunction in 62% of patients with COVID-19. RV dysfunction is increased RV afterload caused by pulmonary thrombosis or embolism [10]. RV enlargement (41%) and RV dysfunction (27%) were also associated with elevated D-dimer and C-reactive protein levels. At the same time, LV function was hyperdynamic or normal in 89% of patients [11, 12].

SARS-CoV-2 virus can induce a range of complications, including thrombotic microangiopathy, venous thromboembolism, endothelial dysfunction, and impaired pulmonary hemodynamics, which can lead to increased pressure in the pulmonary artery. Echocardiographic evaluations in hospitalized patients with COVID-19 reveal the presence of PH in approximately 12-13% of cases. In addition, coronavirus disease exacerbates concomitant PH that is accompanied by chronic congestive heart failure or chronic obstructive pulmonary disease. PH, in turn, worsens the course of the disease and increases the risk of complications [13].

Histological studies have established that the walls of pulmonary vessels were thickened in patients who died from COVID-19, which was not observed in those who died from the severe acute respiratory syndrome (SARS) in 2002-2004 or from the H1N1 flu (2009-2010). Such vascular changes are important for the formation of PH [14].

Echocardiography is useful for detecting the accumulation of pericardial effusion and cardiac tamponade, as it was described in the case of a 47-year-old woman with COVID-19 [15]. There is evidence that COVID-19 increases the risk of acute myocardial infarction or ischemic stroke by destabilizing atherosclerotic plaque in blood vessels [16]. In critically ill patients with myocarditis, myopericarditis, stress cardiomyopathy, or myocardial infarction, a cardiogenic shock could develop occasionally [17]. A fatal case of a severe course of COVID-19 in a 63-year-old man complicated by fulminant myocarditis with a sharp decrease in LV systolic function and cardiogenic shock was described. In this patient, the echocardiography showed decreased left ventricular ejection fraction associated with elevated troponin (11.37 g/L) and a very high level of IL-6 (272.40 pg/mL) [18]. In many cases of COVID-19 (36.54%), echocardiography results led to therapeutic changes [19].

Thus, numerous studies indicate the importance of echocardiography in managing hospitalized patients, enabling the diagnosis of concurrent cardiac pathologies and informing necessary adjustments in treatment strategies [20]. However, a persistent challenge is differentiating between preexisting cardiac anomalies and those induced by COVID-19 infection [21]. This study aimed to establish echocardiogram abnormalities and identify the most important prognostic factors associated with severe and fatal COVID-19 outcomes.

## MATERIAL AND METHODS

### Study design and participants

This retrospective cohort study is part of a larger project conducted by Ivano-Frankivsk National Medical University, titled "The course of infectious diseases against the background of concomitant pathology, combined chronic infections and invasions, correction of treatment" (state registration number: 0119U100571, implementation dates: 2021 – 2023). We analyzed echocardiographic data from 194 patient records at the Regional Clinical Infectious Diseases Hospital of Ivano-Frankivsk, Ukraine. The study included 100 cases of moderate severity, 34 severe cases without fatalities (with respiratory failure (RF) grades I to III), and 60 cases of severe RF (grades III to IV) that resulted in death. The inclusion criteria for each group of patients were the corresponding severity and outcomes of the disease. Exclusion criteria were lung cancer and death not caused by respiratory failure.

### Clinical assessment

Patient diagnoses, including respiratory failure (RF), concomitant pathologies, echocardiographic data, and body mass index (BMI) were extracted from medical records. Echocardiograms were performed using a Toshiba Nemio XG machine, employing apical and parasternal long-axis B- and M-mode imaging with a 3.5 MHz sector transducer. The protocol adhered to the joint guidelines of the American Society of Echocardiography and the European Association of Cardiovascular Imaging [22]. CHF was diagnosed according to the New York Heart Association 1994 criteria. Pulmonary hypertension was determined by the peak tricuspid regurgitation velocity according to the 2022 ESC/ERS guidelines [23], with velocities ≤2.8 m/s rated as low (degree 1), between 2.9 and 3.4 m/s as intermediate (degree 2), and >3.4 m/s as high (degree 3). BMI was calculated to categorize obesity: class I for BMI between 30 to 35, class II for BMI between 35 to 40, and class III for BMI over 40, following Center for Disease Control and Prevention (CDC) guidelines. Hypertension stages I, II, and III were determined per the ESC/ESH guidelines for managing arterial hypertension [24].

The severity of COVID-19 was assessed following the World Health Organization clinical management guidance for COVID-19 [25]. The classification of Adult Acute Respiratory Distress Syndrome was based on the widely recognized 2012 Berlin Definition [26].

### Statistical analysis

We used Pearson's chi-square test (χ2) to compare the frequency of presented pathologies in groups of patients and Student's t-test to compare the mean age of patients. For this reason, an online statistic calculator was used, and significant differences were considered when p<0.05 [27].

## RESULTS

### Clinical characteristics and comorbidities of patients with moderate and severe COVID-19 with a favorable outcome

The study included 100 patients with moderate COVID-19, comprising 49 men and 51 women, with an age range of 31 to 80 years and a mean age of 60.78. These patients had X-ray and ultrasound signs of bilateral pneumonia affecting 20% to 50% of the lung fields, and there were no signs of RF. The duration of hospital stay ranged from 8 to 29 days, with an average of 14.9±1.48 days. Medical charts indicated the presence of concomitant pathologies: diffuse cardiosclerosis (54.0%), hypertension (32.0%), obesity (10.0%), CHF (4.0%), and type 2 diabetes (4.0%) (Table 1).

**Table 1:** Comorbidities in patients with moderate and severe COVID-19 with a favorable outcome, N=134 (%)

Comorbidities	Moderate, N=100		Severe, N=34		χ^2^	p-value
	N	%	N	%		
Diffuse cardiosclerosis	54	54.0	25	73.5	3.999	0.046
Postinfarction cardiosclerosis	0	0.0	1	2.9	2.963	0.086
FCHF FC I	2	2.0	3	8.8	5.656	0.018
FCHF FC II	1	1.0	5	14.7	11.44	<0.001
FCHF FC III	1	1.0	0	0.0	0.343	0.556
Total number of CHF	4	4.0	8	23.5	11.869	<0.001
Hypertension I	1	1.0	0	0.0	0.343	0.556
Hypertension II	31	31.0	22	64.7	12.057	<0.001
Total number of hypertension	32	32.0	22	64.7	11.281	<0.001
Encephalopathy	0	0.0	1	2.9	2.963	0.086
Type 2 diabetes, compensated	4	4.0	3	8.8	1.192	0.275
Diabetes 2, decompensated	0	0.0	7	20.6	21.723	<0.001
Total number of type 2 diabetes	4	4.0	10	29.4	17.512	<0.001
Obesity I	5	5.0	1	2.9	0.251	0.617
Obesity II	4	4.0	7	20.6	9.266	0.002
Obesity III	1	1.0	2	5.9	2.763	0.096
Obesity (total number)	10	10.0	10	29.4	7.530	0.007
Artificial pacemaker	0	0.0	1	2.9	2.963	0.086
Stenting of coronary arteries	0	0.0	1	2.9	2.963	0.086
Chronic pulmonary heart diseases	0	0.0	1	2.9	2.963	0.086
Ventricular extrasystoles	0	0.0	1	2.9	2.963	0.086
Atrial fibrillation	0	0.0	1	2.9	2.963	0.086
Kidney stone disease	0	0.0	2	5.9	5.971	0.015
Anxiety disorder	0	0.0	1	2.9	2.963	0.086
Anemia	0	0.0	1	2.9	2.963	0.086

χ^2^ – Pearson's chi-square test; p - statistical significance

The severe group with favorable outcomes included 34 patients—19 men (55.9%) and 15 women (44.1%) - ranging in age from 44 to 81 years, with a mean age of 63.9±1.7 years. The average age of these patients did not statistically differ from those with moderate severity (60.78±1.38, t=1.215, p>0.05). Patients were hospitalized for 12-36 days (22.2±1.0 days on average). X-ray and ultrasound of the lungs showed bilateral pneumonia with damage of 50% - 80% of the lung fields. Respiratory insufficiency was categorized as follows: Grade I in 14 patients (41.2%), Grade II in 13 patients (38.2%), and Grade III in 7 patients (20.6%). A comparison of comorbidities with the moderate COVID-19 group showed a higher incidence of diffuse cardiosclerosis (73.5% *vs*. 54.0%, p=0.046) and CHF (23.5% *vs*. 4.0%, p<0.001), particularly CHF FC II (14.7% *vs*. 1.0%, p<0.001). Hypertension stages I and II (64.7% *vs*. 32.0%, p<0.001), type 2 diabetes (29.4% *vs*. 4.0%, p<0.001), in the decompensation stage (20.6 *vs*. 0.0%, p<0.001) and all classes of obesity (26.5% *vs*. 10.0%, p<0.001), especially II (20.6 *vs*. 4.0%, p=0.002) were also more prevalent in the severe group.

Less common pathologies, such as the presence of an artificial pacemaker, coronary stent, and other cardiovascular and metabolic conditions, were individually not so frequent but collectively significant when compared to the moderate group (26.5% *vs*. 0.0%, χ^2^ = 33.811, p<0.001) (Table 1).

### Echocardiographic findings in patients with moderate and severe COVID-19 with a favorable outcome

In moderate patients, a number of abnormalities were revealed by the echocardiography: LV hypertrophy in 22.0%, diffuse cardiosclerosis in 36.0%, aortic aneurysm in 1.0%, stenosis of the aortic opening in 2.0% and AV insufficiency in 3.0%. Compaction and fibrosis were also observed in the aortic valve in 11.0% of cases, the mitral valve (MV) in 21.0%, and the tricuspid valve (TC). These pathologies are more likely preexisting conditions rather than directly associated with COVID-19. Blood flow regurgitation was also noted in the mitral valve (18.0%), aortic valve (3.0%), and tricuspid valve (11.0%). A significant reduction in LV ejection fraction was observed: 7.0% of patients showed a pronounced decrease to less than 28%, and 3.0% experienced a reduction to less than 50%. Grade I diastolic dysfunction of the LV was recorded in 8.0% of the cases (Table 2). These changes suggest decreased LV relaxation, likely due to preexisting conditions such as hypertrophy and cardiosclerosis. However, these issues may be exacerbated by the systemic immune inflammatory response associated with COVID-19. Furthermore, valve regurgitation, preexisting in some patients, could worsen due to weakened myocardial contractile function caused by the virus [28-30].

**Table 2:** Transthoracic echocardiography findings in patients with moderate and severe COVID-19 with a favorable outcome, N=134 (%)

Echocardiography data	Moderate, N=100		Severe, N=34		χ^2^	p-value
	N	%	N	%		
LV hypertrophy	22	22.0	14	41.2	4.749	0.030
Cardiosclerosis	36	36.0	25	73.5	14.410	<0.001
Aortic aneurysm	1	1.0	1	2.9	0.650	0.421
AV stenosis	2	2.0	2	5.9	1.321	0.250
AV dilatation	3	3.0	2	5.9	0.587	0.444
AV compaction and fibrosis	11	11.0	12	35.3	10.533	0.001
Regurgitation on AV	3	3.0	8	23.5	14.192	<0.001
MV compaction and fibrosis	21	21.0	14	41.2	5.353	0.021
Regurgitation on MV	18	18.0	10	29,4	1.999	0.157
RV dilation	7	7.0	8	23.5	6.974	0.008
TV compaction and fibrosis	6	6.0	5	14.7	5.353	0.111
Regurgitation on TV	11	11.0	8	23.5	3.27	0.070
PA dilation (2.1-2.5 cm)	18	18.0	12	35.3	4.367	0.037
Pulmonary hypertension						
1 degree	7	7.0	5	14.7	1.848	0.174
2 degree	4	4.0	5	14.7	4.642	0.031
PH total number	10	10.0	10	29.4	7.530	0.006
Pericardial compaction	16	16.0	9	26.5	1.833	0.176
Exudative pericarditis	8	8.0	7	20.6	4.045	0.044
Reduced systole fraction of LV (≤28%)	7	7.0	8	23.5	6.974	0.008
Reduced ejection fraction of LV (≤50%)	3	3.0	5	14.7	6.193	0.013
Grade I LV diastolic dysfunction	8	8.0	7	20.6	4.045	0.044

χ^2^ – Pearson's chi-square test; p - statistical significance

Other inflammatory changes identified were a thickening of the pericardium in 16.0% of patients and a moderately increased amount of pericardial fluid in 8.0%. PH was detected in 10.0% of patients (first degree – 7.0% and second degree – 4.0%) (Table 2). It could arise or intensify against the background of lesions of pulmonary capillaries (microthrombi, vasculitis) and increased peripheral resistance to blood flow in the small circle of blood circulation [31, 32].

Echocardiography in patients with severe COVID-19 revealed more frequent and pronounced cardiac abnormalities compared to those with moderate disease severity (Table 2). Thus, diffuse cardiosclerosis was significantly more common in severe cases (73.5%) compared to moderate cases (36.0%, p<0.001). Similarly, fibrosis and compaction of the AV were observed in 35.3% of severe cases *versus* 11.0% in moderate cases (p=0.001), and valve regurgitation was seen in 23.5% of severe cases, significantly higher than the 3.0% in moderate cases (p<0.001). The MV also showed more fibrosis and compaction in severe cases (41.2%) compared to moderate cases (21.0%, p=0.021). There were changes in the dimensions of the walls and chambers of the ventricles: LV hypertrophy in 41.1% *vs*. 22.0%, respectively, p=0.030; RV dilatation in 23.5% *vs*. 7.0%, respectively, p=0.008; PA dilatation (2.1 - 2.5 cm in diameter) in 35.3% *vs*. 18.0%, respectively, p=0.037. A violation of the contractile function of the LV was observed more often in severe patients than in moderate patients. Thus, Impairments in LV contractile function were more frequent in severe cases, with a decrease in systolic fraction observed in 23.5% (*versus* 7.0% in moderate cases, p=0.008), a decrease in ejection fraction in 14.7% (*versus* 3.0%, p=0.013), and grade I diastolic dysfunction of LV (hypertrophic type) in 20.6% (*versus* 8.0%, p=0.044). In severe patients, PH was more prevalent (29.4%) compared to moderate cases (10.0%, p=0.006), with a higher incidence of degree 2 PH in severe patients (14.7% *vs*. 4.0% in moderate cases, p=0.031). A compaction of pericardium with exudation was also found more often in patients with a severe course of COVID-19 (20.6 % *vs*. 8.0%, respectively, p=0.044) (Table 2).

### Clinical characteristics and comorbidities of patients with severe COVID-19 with a fatal outcome

The group of patients with a severe course and fatal outcome consisted of 60 people (51.7% men and 48.3% women) aged 33-89 years (average age was 67.1±1.51 years). The average age was not statistically different from patients who survived severe COVID-19 (average age 63.9±1.7 years, t=1.407, p>0.05) but was significantly higher than those with moderate severity (average age 60.78±1.38 years, t=2.965, p<0.01). These patients were hospitalized for 1-46 days (average 14.47±1.51 days) and commonly exhibited bilateral pneumonia, affecting 75%-90% of lung fields, along with III-degree respiratory failure. Medical records indicated several concurrent cardiovascular and metabolic diseases. In patients with a fatal outcome, diffuse cardiosclerosis (90.0% *vs*. 73.5%, p=0.03) and CHF (61.7% *vs*. 23.5%, p=0.0004), particularly functional class II CHF (41.7% *vs*. 14.7%, p=0.008), were more prevalent compared to those with a favorable outcome. The frequency of other conditions like hypertension, diabetes, obesity, and others was similar in both groups (Table 3). On the other hand, hypertension stage III was observed in only four patients who had a fatal outcome, and a post-stroke condition was noted in one patient. Such data indirectly indicate a more severe clinical course of hypertension (Table 3).

**Table 3:** Comorbid pathologies in patients with severe COVID-19 with favorable and fatal outcomes, N=94

Pathologies	Fatal outcomes, N=60		Favorable outcome, N=34		χ^2^	p-value
	N	%	N	%		
Diffuse cardiosclerosis	54	90.0	25	3.5	4.389	0.036
Postinfarction cardiosclerosis	2	3.3	1	2.9	0.011	0.918
Stenting of coronary arteries	1	1.7	1	2.9	0.169	0.681
Artificial pacemaker	1	1.7	1	2.9	0.169	0.681
Pulmonary heart disease	1	1.7	1	2.9	0.169	0.681
Aortic stenosis	1	1.7	0	0.0	0.573	0.450
Ventricular extrasystoles	2	3.3	1	2.9	0.011	0.918
Atrial fibrillation	2	3.3	1	2.9	0.011	0.918
CHF I, FC I	5	8.3	3	8.8	0.007	0.935
CHF IIA, FC II	25	41.7	5	14.7	7.260	0.008
CHF IIB, FCIII	5	8.3	0	0.0	2.993	0.084
CHFIII, FCIV	2	3.3	0	0.0	1.158	0.282
CHF (total number)	37	61.7	8	23.5	12.649	0.0004
Hypertension II	26	43.3	18	52.9	0.805	0.370
Hypertension III	4	6.7	0	0.0	2.367	0.124
Hypertension (total number)	30	50.0	22	64.7	1.899	0.169
Post-stroke state	1	1.7	0	0.0	0.573	0.450
Dyscirculatory encephalopathy	3	5.0	1	2.9	0.226	0.635
Compensated type 2 diabetes	6	10.0	3	8.8	0.035	0.853
Decompensated type 2 diabetes	20	33.3	7	20.6	1.722	0.190
Type 2 diabetes (total number)	26	43.3	10	29.4	2.641	0.105
Type 1 diabetes	1	1.7	0	0.0	0.573	0.450
Obesity I	4	6.7	1	2.9	0.598	0.440
Obesity II	10	16.7	7	20.6	0.225	0.636
Obesity III	6	10.0	2	5.9	0.473	0.492
Obesity (total number)	20	33.3	10	29.4	0.154	0.696
Non-Hodgkin’s lymphoma	1	1.7	0	0.0	0.573	0.450
Myeloma disease	1	1.7	0	0.0	0.573	0.450
HIV infection, wasting syndrome	1	1.7	0	0.0	0.573	0.450
Alcoholic epilepsy	1	1.7	0	0.0	0.573	0.450
Cerebral arachnoiditis	1	1.7	0	0.0	0.573	0.450
Anxiety disorder	1	1.7	1	2.9	0.169	0.681
Organic poly-syndrome	1	1.7	0	0.0	0.573	0.450
Parkinson’s disease	1	1.7	0	0.0	0.573	0.450
Anemia	2	3.3	1	2.9	0.011	0.918
Liver cysts	2	3.3	0	0.0	1.158	0.282
Kidney cysts	2	3.3	0	0.0	1.158	0.282
Kidney stone disease	1	1.7	2	5.9	1.248	0.264
Chronic renal failure I-II	3	5.0	0	0.0	1.756	0.186

χ^2^ – Pearson's chi-square test; p - statistical significance

### Echocardiographic findings in patients with severe COVID-19 with fatal outcomes

Echocardiographic analysis revealed notable differences between patients with severe COVID-19 who had fatal outcomes and those with favorable outcomes. In patients with fatal outcomes, aortic arch expansion was observed in 11.7%, compared to none in the favorable outcome group (p=0.039). Additionally, compaction and fibrosis of the AV were more prevalent in fatal cases (58.3%) than in severe cases with favorable outcomes (35.3%, p=0.032). A significant decrease in ejection fraction was also more common in fatal cases (36.7% *vs*. 14.7%, p=0.024). Various types of myocardial dysfunction were observed, including grade I diastolic dysfunction of the LV (hypertrophic type) in 26.7% of fatal cases. Other dysfunctions like grade II diastolic dysfunction (pseudonormal type), LV hypokinesia, paradoxical movement of the LV, and dyskinesia of the interventricular septum were seen in isolated cases, but these did not show statistical differences between the two groups. The overall incidence of myocardial dysfunction was significantly higher in patients with a critically severe course and a fatal outcome (51.7% *vs*. 29.4%, respectively, p=0.037). There was a trend towards an increase in the frequency of pulmonary hypertension (48.3% *versus* 29.4%, respectively, p=0.074) (Table 4).

**Table 4:** Transthoracic echocardiography findings in patients with severe COVID-19 with favorable and fatal outcomes, N=94

Echocardiography data	Fatal outcome, N=60		Favorable outcome, N=34		χ^2^	p-value
	N	%	N	%		
LV hypertrophy	29	48.3	14	41.2	0.448	0.503
LV dilatation	1	1.7	0	0.0	0.573	0.450
Cardiosclerosis	41	68.3	25	73.5	0.280	0.597
Dilatation of aorta	8	13.3	1	2.9	2.707	0.100
**Aneurysm of LV**	1	1.7	0	0.0	0.573	0.450
AV stenosis	7	11.7	2	5.9	0.839	0.360
AV dilatation	7	11.7	2	5.9	0.839	0.360
AV Consolidation and fibrosis	35	58.3	12	35.3	4.608	0.032
Regurgitation on AV	9	15.0	8	23.5	1.066	0.302
Consolidation and fibrosis MV	19	31.7	14	41.2	0.862	0.353
Regurgitation on MV	23	38.3	10	29.4	0.758	0.384
RV dilation	18	30.0	8	23.5	0.454	0.501
RA dilatation	3	5.0	0	0.0	1.756	0.186
TV consolidation and fibrosis	9	15.0	5	14.7	0.001	0.970
TV regurgitation	12	20.0	8	23.5	0.161	0.688
PA dilatation (2.1-2.5 cm)	20	33.3	12	35.3	0.037	0.848
Pulmonary hypertension: 1 degree 2 degree 3 degree	29 13 15 1	48.3 21.7 25.0 1.7	10 5 5 0	29.4 14.7 14.7 0.0	3.201 0.679 1.373 0.688	0.074 0.410 0.241 0.407
Pericardial compaction	18	30.0	9	26.5	0.132	0.717
Exudative pericarditis	8	13.3	7	20.6	0.852	0.357
Reduced LV systole fraction (≤28%)	12	20.0	8	23.5	0.161	0.688
Reduced LV ejection fraction (≤55%)	22	36.7	5	14.7	5.112	0.024
Grade I LV Diastolic dysfunction	16	26.7	7	20.6	0.434	0.510
Grade II LV diastolic dysfunction	4	6.7	2	5.9	0.022	0.881
LV hypokinesia	2	3.3	1	2.9	0.011	0.917
LV diastolic dyskinesia	3	5.0	0	0.0	1.756	0.186
Paradoxical movement of the LV	2	3.3	0	0.0	1.158	0.282
Dyskinesia of the interventricular septum	4	6.7	0	0.0	2.367	0.124
All types of myocardial dysfunction (total number)	31	51.7	10	29.4	4.371	0.037

χ^2^ – Pearson's chi-square test; p - statistical significance

## DISCUSSION

The study showed that the average age of hospitalized patients was 60 years or older, with the risk of a fatal outcome more pronounced at an average age of 67.1±1.51 years. Comorbidities such as obesity class II (BMI > 35.0-39.9), type 2 diabetes, hypertension, chronic heart pathology, and CHF were often found in patients with severe COVID-19. These underlying chronic diseases are generally recognized as risk factors for severe diseases. Echocardiographic examinations revealed a broad spectrum of cardiac abnormalities, indicating underlying myocardial pathologies. The most common underlying disease was diffuse cardiosclerosis, which presented in 73.5% of patients with a favorable outcome and 90.0% of patients with a fatal outcome. Further echocardiographic findings included aortocardiosclerosis features like aortic arch expansion, fibrosis, and thickening and regurgitation of the aortic mitral and tricuspid valves. Such data reflects age-related changes in the heart. Heart failure, a significant outcome of cardiosclerosis, was more frequently observed in severe cases than in moderate cases (23.53% *vs*. 4.0%). Undoubtedly, HF was the most important aggravating factor and had a direct effect on increasing the frequency of adverse outcomes. Background chronic heart failure may worsen or can be a consequence of acute myocarditis under the influence of SARS-CoV-2 [5-7]. Generally, HF was detected more than twice as often in patients with unfavorable outcomes (58.3% *vs*. 23.5%, respectively). Furthermore, functional class II chronic heart failure (FCII CHF) was three times more common in patients with fatal outcomes (41.7% *vs*. 14.7%).

The echocardiographic confirmation of HF included a reduction in the LV ejection fraction, observed in 36.7% of patients with fatal outcomes and 14.7% of patients with favorable outcomes. There were cases of diastolic dysfunction of the LV grade I (prolongation of the interval of slow blood filling the LV), which occurred in 20.6% of severe patients with favorable outcomes and 26.7% in fatal cases. LV diastolic dysfunction grade I may also increase pressure in the left atrium and pulmonary vessels [33].

The increase in the frequency of PH was quite typical for patients with massive lung damage (70% - 90%). In our study, PH was present in 48.3% of patients with fatal COVID-19 outcomes. While PH in moderate COVID-19 cases may be an underlying condition, its increased prevalence in severe cases, particularly those with fatal outcomes, is likely attributed to extensive lung damage [14, 34]. This increase in PH could be driven by mechanisms such as endothelial dysfunction [14, 34], microangiothrombosis in alveolar capillaries [35], and a reduction in nitric oxide levels [36], all contributing to increased resistance in the pulmonary circulation.

The presence of PH further complicates pulmonary gas exchange due to shunting in the pulmonary capillaries [37] and adds to the hemodynamic burden on the right side of the heart. This was evidenced by the observed dilation of the right ventricle in 18.0% of fatal cases, the right atrium in 5.0%, and the pulmonary artery in 33.3%. These findings suggest a possible association between post-COVID pulmonary gas exchange disorders, cardiovascular complications, and the development of PH [38, 39].

Endothelial dysfunction has a very important role in the progression of severe COVID-19, particularly observed in patients with type 2 diabetes mellitus [40] and hypertension [41]. It has been established that endothelial dysfunction is a universal pathogenetic factor of multiorgan lesions in patients with COVID-19. In patients with HF, there is an increased expression of angiotensin-converting enzyme 2 receptors (ACE2), which could explain the exacerbated severity of COVID-19 in these individuals [42, 43]. The pericardial thickening without/with exudation in the pericardial sac is likely associated with a systemic inflammatory response and cytokine proinflammatory storm [44-47].

The study corroborated well-established risk factors for severe COVID-19, which include an average age over 60 years (in our study, the average age was 63.9±1.7 years), CHF, hypertension, type 2 diabetes mellitus, and obesity class II. These risk factors were consistent among participants with severe disease. Moreover, the most critical factors leading to fatal outcomes included an older average age of 67.1 years, CHF functional class II, reduced ejection and systolic fractions of the left ventricle, multiple LV dysfunctions, and pulmonary hypertension.

One of the strengths of the study is the analysis of echocardiography data and its correlation with the severity of the disease, patient outcomes, and age. One limitation is the absence of inclusion and exclusion criteria based on the different management protocols for COVID-19, which could influence patient outcomes. Additionally, the frequent use of echocardiography to monitor disease progression could reveal valuable insights into the dynamic nature of the illness.

## CONCLUSION

The clinical and echocardiographic examinations conducted in this study identified critical cardiological risk factors that contribute to the severe progression of COVID-19 and increase the likelihood of a fatal outcome. These include chronic heart failure (CHF FC II), reduced ejection and systolic fraction of the LV, and various dysfunctions of both the left and right ventricles.

## Data Availability

Further data is available from the corresponding author upon reasonable request.

## Personal thanks

We express our gratitude to the administration of the Communal Non-Profit Enterprise Regional Infectious Diseases Hospital of the Ivano-Frankivsk Regional Council and the medical staff for treating patients and assisting in conducting research.

## References

[1] Mehraeen E, Karimi A, Barzegary A, Vahedi F (2020). Predictors of mortality in patients with COVID-19–a systematic review. Eur J Integr Med.

[2] Tylishchak Z, Pryshliak O, Skrypnyk N, Boichuk O (2023). Coronavirus disease (COVID-19) in patients with type 2 diabetes mellitus: clinical and laboratory peculiarities. RJDNMD.

[3] Giustino G, Croft LB, Stefanini GG, Bragato R (2020). Characterization of Myocardial Injury in Patients With COVID-19. J Am Coll Cardiol.

[4] Bhatt AS, Jering KS, Vaduganathan M, Claggett BL (2021). Clinical Outcomes in Patients with Heart Failure Hospitalized With COVID-19. JACC Heart Fail.

[5] Bader F, Manla Y, Atallah B, Starling RC (2021). Heart failure and COVID-19. Heart Fail Rev.

[6] Cordero A, García-Gallego CS, Bertomeu-González V, Fácila L (2021). Mortality associated with cardiovascular disease in patients with COVID-19. REC: Cardioclinics.

[7] Tomasoni D, Inciardi RM, Lombardi CM, Tedino C (2020). Impact of heart failure on the clinical course and outcomes of patients hospitalized for COVID-19 Results of the Cardio-COVID-Italy multicentre study. Eur J Heart Fail.

[8] Corica B, Marra AM, Basili S, Cangemi R (2021). Prevalence of right ventricular dysfunction and impact on all-cause death in hospitalized patients with COVID-19: a systematic review and meta-analysis. Sci Rep.

[9] Oktaviono YH, Mulia EP B, Luke K, Nugraha D (2021). Right ventricular dysfunction and pulmonary hypertension in COVID-19: a meta-analysis of prevalence and its association with clinical outcome. Arch Med Sci.

[10] Norden N, Lundin EO, Hagberg E, Gao SA (2021). Cardiac involvement in critically ill and mechanically ventilated patients with COVID-19-a prospective, observational echocardiographic study. Am J Cardiovasc Dis.

[11] Mahmoud-Elsayed HM, Moody WE, Bradlow WM, Khan-Kheil AM (2020). Echocardiographic Findings in Patients With COVID-19 Pneumonia. Can J Cardiol.

[12] Schott JP, Mertens AN, Bloomingdale R, O'Connell TF (2020). Transthoracic echocardiographic findings in patients admitted with SARS-CoV-2 infection. Echocardiography.

[13] Castiglione L, Droppa M (2022). Pulmonary Hypertension and COVID-19. Hamostaseologie.

[14] Suzuki YJ, Nikolaienko SI, Shults NV, Gychka SG (2021). COVID-19 patients may become predisposed to pulmonary arterial hypertension. Med Hypotheses.

[15] Hua A, O'Gallagher K, Sado D, Byrne J (2020). Life-threatening cardiac tamponade complicating myo-pericarditis in COVID-19. Eur Heart J.

[16] Modin D, Claggett B, Sindet-Pedersen C, Lassen MCH (2020). Acute COVID-19 and the Incidence of Ischemic Stroke and Acute Myocardial Infarction. Circulation.

[17] Shah PB, Welt FGP, Mahmud E, Phillips A (2020). Triage considerations for patients referred for structural heart disease intervention during the coronavirus disease 2019 (COVID-19) pandemic: An ACC /SCAI Consensus Statement. J Am Coll Cardiol Intv.

[18] Zeng JH, Liu YX, Yuan J, Wang FX (2020). First case of COVID-19 complicated with fulminant myocarditis: a case report and insights. Infection.

[19] Bagate F, Masi P, d'Humières T, Al-Assaad L (2021). Advanced echocardiographic phenotyping of critically ill patients with coronavirus-19 sepsis: a prospective cohort study. J Intensive Care.

[20] Jain SS, Liu Q, Raikhelkar J, Fried J (2020). Indications for and Findings on Transthoracic Echocardiography in COVID-19. J Am Soc Echocardiogr.

[21] Task Force for the management of COVID-19 of the European Society of Cardiology (2022). European Society of Cardiology guidance for the diagnosis and management of cardiovascular disease during the COVID-19 pandemic: part 1-epidemiology, pathophysiology, and diagnosis. Eur Heart J.

[22] Lang RM, Badano LP, Mor-Avi V, Afilalo J (2015). Recommendations for cardiac chamber quantification by echocardiography in adults: an update from the American Society of Echocardiography and the European Association of Cardiovascular Imaging. J Am Soc Echocardiogr.

[23] Humbert M, Kovacs G, Hoeper MM, Badagliacca R, ESC/ERS Scientific Document Group 2022 (2023). ESC/ERS Guidelines for the diagnosis and treatment of pulmonary hypertension. Eur Respir J.

[24] Williams B, Mancia G, Spiering W, Agabiti Rosei E, ESC Scientific Document Group (2018). ESC/ESH Guidelines for the management of arterial hypertension. Eur Heart J.

[25] World Health Organization (2021). Living guidance for clinical management of COVID-19. https://www.who.int/publications/i/item/WHO-2019-nCoV-clinical-2021-2.

[26] Nickson C (2020). Acute Respiratory Distress Syndrome Definitions Life in the fastlane Nov 3. https://litfl.com/acute-respiratory-distress-syndrome-definitions/.

[27] Social sciences statistics Chi Square Calculator for 2x2. https://www.socscistatistics.com/tests/chisquare/default.aspx.

[28] Italia L, Ingallina G, Napolano A, Boccellino A (2021). Subclinical myocardial dysfunction in patients recovered from COVID-19. Echocardiography.

[29] Bieber S, Kraechan A, Hellmuth JC, Muenchhoff M (2021). Left and right ventricular dysfunction in patients with COVID-19-associated myocardial injury. Infection.

[30] Aggarwal G, Cheruiyot I, Aggarwal S, Wong J (2020). Association of Cardiovascular Disease With Coronavirus Disease 2019 (COVID-19) Severity: A Meta-Analysis. Curr Probl Cardiol.

[31] Widrich J, Shetty M (2023). Physiology, Pulmonary Vascular Resistance. https://www.ncbi.nlm.nih.gov/books/NBK554380/.

[32] Kulik TJ (2012). Pulmonary blood flow and pulmonary hypertension: Is the pulmonary circulation flowophobic or flowophilic?. Pulm Circ.

[33] Tudoran M, Tudoran C, Lazureanu VE, Marinescu AR (2021). Alterations of Left Ventricular Function Persisting during Post-Acute COVID-19 in Subjects without Previously Diagnosed Cardiovascular Pathology. J Pers Med.

[34] Mishra A, Lal A, Sahu KK, George AA (2020). An Update on Pulmonary Hypertension in Coronavirus Disease-19 (COVID-19). Acta Biomed.

[35] Asakura H, Ogawa H (2021). COVID-19-associated coagulopathy and disseminated intravascular coagulation. Int J Hematol.

[36] Abraham GR, Kuc RE, Althage M, Greasley PJ (2022). Endothelin-1 is increased in the plasma of patients hospitalised with Covid-19. J Mol Cell Cardiol.

[37] Katsoularis I, Fonseca-Rodríguez O, Farrington P, Lindmark K, Fors Connolly AM (2021). Risk of acute myocardial infarction and ischaemic stroke following COVID-19 in Sweden: a self-controlled case series and matched cohort study. Lancet.

[38] Tudoran C, Tudoran M, Lazureanu VE, Marinescu AR (2021). Evidence of Pulmonary Hypertension after SARS-CoV-2 Infection in Subjects without Previous Significant Cardiovascular Pathology. J Clin Med.

[39] Tudoran C, Tudoran M, Lazureanu VE, Marinescu AR (2021). Factors Influencing the Evolution of Pulmonary Hypertension in Previously Healthy Subjects Recovering from a SARS-CoV-2 Infection. J Clin Med.

[40] Hadi HA, Suwaidi JA (2007). Endothelial dysfunction in diabetes mellitus. Vasc Health Risk Manag.

[41] Gallo G, Volpe M, Savoia C (2022). Endothelial Dysfunction in Hypertension: Current Concepts and Clinical Implications. Front Med (Lausanne).

[42] Xu Sw, Ilyas I, Weng Jp (2023). Endothelial dysfunction in COVID-19: an overview of evidence, biomarkers, mechanisms and potential therapies. Acta Pharmacol Sin.

[43] Katsoularis I, Fonseca-Rodríguez O, Farrington P, Lindmark K, Fors Connolly AM (2021). Risk of acute myocardial infarction and ischaemic stroke following COVID-19 in Sweden: a self-controlled case series and matched cohort study. Lancet.

[44] Li G, Hu R, Gu X (2020). A close-up on COVID-19 and cardiovascular diseases. Nutr Metab Cardiovasc Dis.

[45] Kang Y, Chen T, Mui D, Ferrari V (2020). Cardiovascular manifestations and treatment considerations in COVID-19. Heart.

[46] Soewono KY, Raney KC, Sidhu MS (2021). Pericarditis with pericardial effusion as a delayed complication of COVID-19. Proc (Bayl Univ Med Cent).

[47] Deana C, Vetrugno L, Fabris M, Curcio F (2022). Pericardial Cytokine "Storm" in a COVID-19 Patient: the Confirmation of a Hypothesis. Inflammation.

